# Target achievement and cardiovascular event rates with Lomitapide in homozygous Familial Hypercholesterolaemia

**DOI:** 10.1186/s13023-018-0841-3

**Published:** 2018-06-20

**Authors:** Dirk J. Blom, Marina Cuchel, Miranda Ager, Helen Phillips

**Affiliations:** 1 Division of Lipidology, Department of Medicine, University of Cape Town, Cape Town, South Africa; 2 Institute for Translational Medicine and Therapeutics, Cardiovascular Institute, University of Pennsylvania, Philadelphia, PA 19104 USA; 3 Arduvo Ltd, London, UK; 4 Aegerion Pharmaceuticals Ltd, Uxbridge, UK

**Keywords:** (3–10): Homozygous familial hypercholesterolemia, Lomitapide, Number needed to treat, Target, Low-density lipoprotein cholesterol, Major adverse cardiovascular event

## Abstract

**Background:**

Homozygous familial hypercholesterolaemia (HoFH) is characterized by a markedly increased risk of premature cardiovascular (CV) events and cardiac death. Lomitapide reduces low-density lipoprotein cholesterol (LDL-C) levels; however, the probable impact on LDL-C goals and CV events is unknown.

**Methods:**

We used data collected in the first 26 weeks of the lomitapide pivotal phase 3 study (NCT00730236) to evaluate achievement of European Atherosclerosis Society (EAS) LDL-C targets. We used publicly available data reporting major adverse CV events (MACE) rates from other cohorts of HoFH patients to compare event rates for an equivalent number of patient years of exposure (98) in the lomitapide extension trial (NCT00943306).

**Results:**

Twenty-nine patients were included in the phase 3 study. During the first 26 weeks, 15 (51%) and eight (28%) reached LDL-C targets of 100 mg/dL and 70 mg/dL, respectively, at least once. Fourteen (74%) and 11 (58%) of the 19 patients who remained in the extension study after week 126 reached LDL-C targets of 100 mg/dL and 70 mg/dL at least once during the entire study period. Only two MACE were reported in the lomitapide trials (one cardiac death and one coronary artery bypass graft (CABG)) – equivalent to 1.7 events per 1000 patient months of treatment. MACE rates were 21.7, 9.5 and 1.8 per 1000 patient-months respectively in cohorts of HoFH patients pre- and post-mipomersen, and receiving evolocumab. On treatment LDL-C levels were 166, 331 and 286 mg/dL for lomitapide, mipomersen and evolocumab, respectively.

**Conclusions:**

Approximately three quarters and half of patients who took lomitapide for at least 2 years reached LDL-C goals of 100 mg/dL and 70 mg/dL, respectively. There were fewer major CV events per 1000 patient months of treatment in patients taking lomitapide, mipomersen or evolocumab than reported in the mipomersen cohort prior to starting mipomersen. These results support the hypothesis that novel lipid-lowering therapies may reduce CV events in HoFH patients by lowering LDL-C further.

**Trial registration:**

NCT00730236 (registered 8 Aug 2008) and NCT00943306 (registered 22 July 2009).

## Background

Homozygous familial hypercholesterolaemia (HoFH) is a rare genetic condition characterised by markedly elevated low-density lipoprotein-cholesterol (LDL-C) levels, inadequate response to conventional drug therapy and premature-onset cardiovascular disease (CVD) [1]. HoFH is most commonly caused by the occurrence of two LDL receptor *(LDLR*) gene mutations, but can also be caused by mutations in other genes that directly or indirectly act on the LDL/LDL-receptor pathway, including the genes encoding apolipoprotein B *(*apoB; *APOB)*, proprotein convertase subtilisin/kexin type 9 *(PCSK9)*, and LDL-receptor adaptor protein 1 *(LDLRAP1)* [1]. If untreated, patients with HoFH often die from premature CVD in adolescence or early adulthood [2].

The aim of therapy in HoFH is to aggressively reduce LDL-C levels to prevent or delay the onset of premature CV events. The European Atherosclerosis Society (EAS) has suggested that patients with HoFH should be treated to the same LDL-C targets set for other patients requiring lipid-lowering therapy, namely < 100 mg/dL (2.6 mmol/L) for primary prevention, and < 70 mg/dL (1.8 mmol/L) in the presence of clinical atherosclerotic CVD [1]. These targets are not achievable in the majority of HoFH patients with currently available standard therapies that require functioning LDL-receptors, such as statins, ezetimibe [3] and inhibitors of PCSK9 [4].

Lipid apheresis does not require functional *LDLR* and can reduce LDL-C further but is invasive and not universally available. Even with the use of apheresis, targets are often not achieved [5].

Lomitapide is an oral microsomal triglyceride transfer protein inhibitor that reduces the assembly of apoB-containing lipoproteins in the intestine and liver, and therefore does not require functioning LDL-receptors to lower LDL-C levels [6]. Lomitapide was approved as adjunctive therapy in adult patients with HoFH following a small open-label single-arm Phase 3 study. In this study, lomitapide resulted in mean LDL-C reductions of 50.7% (Week 26; safety population; mixed-model repeated measures analysis [7]).

We hypothesise that such large decreases in LDL-C, and in particular the attainment of the EAS recommended LDL-C target levels, may result in a lowered risk of CV events. A paper was recently published modelling the potential improvement in survival and the delay to first major adverse CV event (MACE) in treating patients with HoFH [8]. Formal CV outcome studies to test this hypothesised benefit are not feasible in HoFH due to the relatively small number of affected patients, and ethical concerns about withholding potentially effective therapy from patients with a very severe disorder for prolonged periods of time in a double blind randomised placebo-controlled trial. Therefore, this analysis was conducted to examine the actual benefit achieved in treating patients with HoFH. The objectives of this post hoc, retrospective analysis are to determine the number of HoFH patients receiving lomitapide who reach EAS targets within 6 months, and to compare cardiovascular event rates in patients receiving lomitapide with published data.

## Methods

### Study population

We analysed data from two lomitapide studies: a single-arm, open label, Phase 3 clinical trial of lomitapide in adult patients with HoFH (NCT00730236; the ‘pivotal trial’) and its single-arm extension study (NCT00943306; the ‘extension trial’). Both trials received local institutional review board and regulatory approval. All patients provided written informed consent before participating in the trials, and institutional review boards at each site provided approval of the protocol. Patients enrolled in the pivotal study were required to maintain current lipid-lowering therapy, including apheresis, from 6 weeks before baseline through to at least Week 26. Lomitapide dose was escalated according to safety and tolerability from 5 mg to a maximum of 60 mg/day. The primary endpoint was mean percent change in levels of LDL-C from baseline to Week 26, after which patients remained on lomitapide through to Week 78 for safety assessment. Percent change in LDL-C from baseline was assessed with a mixed linear model [7].

Eligible HoFH patients completing the 78-week Phase 3 pivotal trial were encouraged to enrol into a single-arm extension study where lomitapide was administered daily at the prior maximum tolerated dose (5–60 mg/day) until lomitapide was commercially available in the patient’s country. Patients living in countries where lomitapide was not commercialised were switched to compassionate use [6]. In the safety phase of the pivotal trial (Weeks 26–78) and in the extension trial, changes in the background lipid-lowering therapies were permitted based on physician discretion. Changes to apheresis therapy were only permitted if the patient achieved an LDL-C level ≤ 100 mg/dL [6].

### Cardiovascular event rate comparison

A recent publication by Duell et al. provided a basis from which to calculate cardiovascular event rates in a cohort of HoFH patients receiving conventional lipid-lowering therapy [9]. The authors utilized data from three clinical trials of FH patients receiving mipomersen, plus an open-label study extension phase [9]. Patients who received at least 12 months of mipomersen were included in the analysis. Of the 233 patients analyzed by Duell and colleagues, 51 had HoFH, of whom 23 received at least 12 months of mipomersen therapy [9]. Data from these 23 patients prior to and after starting mipomersen was used for comparative purposes. We also calculated annualised and per 1000 months MACE rates in a HoFH cohort treated with evolocumab based on a recent publication by Raal et al. [10].

For our analysis, we defined MACE as CV death, non-fatal myocardial infarction, coronary revascularization, unstable angina and/or ischemic stroke, to allow for comparisons across trials. We collected MACE data from adverse event reporting during the lomitapide studies, as MACE was not a pre-specified outcome in these studies, and no formal adjudication of MACE during either lomitapide study was made. In the mipomersen studies MACE prevalence pre-treatment was obtained from medical history and case report forms, while MACE incidence was formally adjudicated once patients had commenced treatment with mipomersen [9]. MACE was formally adjudicated in the evolocumab study [10, 11].

## Results

### LDL-C levels in the phase 3 trial and extension trial

The Phase 3 pivotal study enrolled 29 adult men and women with HoFH from 11 centres in four countries (USA, Canada, South Africa, and Italy). Twenty-three of 29 enrolled patients completed both the efficacy phase (26 weeks) and the safety phase (26–78 weeks) [7]. The patients entering the pivotal study were receiving standard of care for HoFH with 93% of patients receiving statins, 76% of patients received ezetimibe while 62% of patients were treated with apheresis. Despite this aggressive therapy, the mean LDL-C at baseline was 336 ± 114 mg/dL (8.70 ± 2.95 mmol/L) [7]. Study visits and LDL-C measurements were always scheduled to take place just prior to the next treatment session in patients receiving apheresis.

Following treatment with lomitapide (median dose 40 mg), LDL-C was reduced by mean of 50.7% (Week 26; safety population; mixed model repeated measures analysis; 40.1% for last observation carried forward analysis; Table 1) [7].

**Table 1:** Mean percent change from baseline in LDL-C levels in pivotal and extension studies

Time point	n	Observed value, mg/dL (SD)	Observed change, mg/dL (SD)	Percent change, % (SD)
Baseline	19	342.8 (125.87)	NA	NA
Week 26	19	158.4 (89.65)	− 184.4 (119.15)	−50.7 (26.77)
Week 78	19	161.2 (59.55)	− 181.7 (110.76)	− 49.0 (19.46)
Week 126	17	188.8 (120.30)	−166.8 (100.28)	−45.5 (31.35)
Week 256	14	143.4 (83.18)	− 224.9 (108.97)	−60.1 (18.51)

*LDL-C* Low-density lipoprotein cholesterol. Values represent percent change in LDL-C levels ± SD from baseline for patients entering the long-term extension study [6]. Background lipid-lowering therapies were fixed until Week 26. Week 78 marks the beginning of the extension phase

Nineteen of 23 patients who completed the pivotal study entered the long-term extension trial (9/19 apheresis) with 16 patients completing all end of study assessments. Lomitapide LDL-C-lowering efficacy was maintained during the extension study and was − 45.5% at Week 126 (Table 1) [6].

In the original cohort (*n* = 29), just over half of patients reached a target LDL-C level < 100 mg/dL at least once by Week 26, and most who remained in the study reached target at least once by Week 78, increasing to 74% by Week 256 (Table 2). The EAS target for patients with clinical atherosclerosis is an LDL-C level of 70 mg/dL or less. Since most of the patients in the studies had CVD (27/29 in the Phase 3 and 17/19 in the extension study), this is the most relevant target for the present analysis. Twenty-eight percent (8/29) of patients reached this stringent target of < 70 mg/dL at least once by Week 26, increasing to 39% of those who remained in the study by Week 78. For the population in the extension trial, 42% had reached the < 70 mg/dL target at least once by Week 126. A further three patients (58%) had reached this target at least once by the end of the extension study, Week 256 (Table 2). When target achievement was assessed over time in the two studies combined, five of the 19 subjects never achieved target, and 14 achieved the LDL-C < 100 mg/dL target once. Of these, 13 reached this target at least on one further occasion during the study. The median number of visits at which LDL-C < 100 mg/dL was achieved during the entire study was 6.0 (range 1–20). The median number of visits at which LDL-C < 70 mg/dL was reached during the entire study was 3.0 (range 1–16). Patients participated in the long-term extension study for variable periods of time, as the extension study usually terminated when lomitapide became commercially available in the participant’s country. The median number of on-treatment visits was 25 (range 17–28).

**Table 2:** Achievement of EAS LDL-C targets at least once during treatment, n (%)

Timeframe	Target	
	< 100 mg/dL (< 2.5 mmol/L)	< 70 mg/dL (< 1.8 mmol/L)
Weeks 0–26^a^ (*n* = 29)	15 (51%)	8 (28%)
Weeks 0–78^a^ (*N* = 23)	16 (70%)	9 (39%)
Weeks 0–126^b^ (*n* = 19	12 (53%)	8 (42%)
Weeks 0–256^b^ (n = 19)	14 (74%)	11 (58%)

*EAS* European Atherosclerosis Society, *LDL-C* Low-density lipoprotein cholesterol

^a^Phase 3 study

^b^Phase 3 and extension study; values represent number of patients and percentage of enrolled population achieving LDL-C targets at least once in the timeframe

As physicians were permitted to amend background lipid-lowering therapies including apheresis (upon achievement of LDL-C < 100 mg/dL) after Week 26, this analysis does not suggest that patients who reached target at least once did so continuously post adjustment of background therapy. Variable compliance or lomitapide dose adjustments in response to treatment-related adverse effects could also compromise maintenance of LDL-C at target.

### Cardiovascular event rate comparison

Only two MACE were reported in the lomitapide trials, both occurring in the long-term extension study (one sudden cardiac death and one coronary artery bypass graft (CABG)). This is equivalent to 1.7 events per 1000 patient months of treatment, or a 2% annualized event rate. Duell et al. [9] recorded 12 MACE in the 23 HoFH patients in the 2 years prior to receiving mipomersen, which equates to 21.7 MACE/1000 months of observation, or a 26% annualized event rate. The MACE rate in the mipomersen cohort decreased to 9.5/ 1000 months following initiation of mipomersen. The MACE rate in the HoFH cohort treated with evolocumab was 1.8/ 1000 months.

Table 3 shows further details of the MACE rates in the cohorts studied.

**Table 3:** Rates for MACE from recent data sources in HoFH

	HoFH background [8]	Mipomersen-treated HoFH [8]	Lomitapide-treated [7]	Evolocumab treated [9]
Number of patients	23	23	19	106
Mean age at baseline	31 years		30.7 years	34 years
Mean baseline LDL-C	455 mg/dL		336 mg/dL	324 mg/dL
Mean LDL-C between 6 and 12 months on treatment^a^	NA	331 mg/dL	166 mg/dL	286 mg/dL
Apheresis	NR	None	62%	32%
CVD at baseline	NR	NR	93%	51%
Number of major CV events^b^	12	4	2	4
Number of patient years	46	35	98	185
Annualized event rate	26.1%	11.4%	2.0%	2.1%
Events/1000 months	21.7	9.5	1.7	1.8

*LDL-C* low-density lipoprotein cholesterol, *NA* not applicable, *NR* not reported [7–9]

^a^Range of follow-up based on data availability: 1-year data for mipomersen, 26-week data for lomitapide, 48-week data for evolocumab, calculated from % reduction reported; CVD, cardiovascular disease [7–9]

^b^MACE was defined as CV death, non-fatal myocardial infarction (MI), unstable angina pectoris and/or ischemic stroke, the evolocumab publication did not include a MACE definition; however, it is believed to be defined as death (CV or non CV), MI, UA, and coronary revascularisation as this is the definition used in the CHMP assessment report for evolocumab [10]

### Adverse events on lomitapide

The present analysis does not include any additional adverse event data beyond those reported for the pivotal Phase 3 trial and the extension study of lomitapide. The Phase 3 study reported gastrointestinal symptoms as the most common adverse event. Experience to date shows that the gastrointestinal symptoms are mostly manageable with dose reductions or temporary treatment interruptions. Four patients experienced elevated aminotransaminase levels of more than five times the upper limit of normal. These resolved after dose reduction or temporary interruption of lomitapide, and no patient permanently discontinued treatment due to liver abnormalities [6]. In common with mipomersen, lomitapide use is associated with increased hepatic fat (hepatic steatosis), with large interindividual variability in the magnitude of increase. The long-term impact of hepatic steatosis associated with lomitapide use is currently unknown. Both the Food and Drug Administration (FDA) and European Medicines Agency (EMA) require lomitapide prescribers to regularly evaluate patients for hepatotoxicity and other adverse effects under risk management programs. Although, adverse events (AEs) may limit the use of lomitapide in some patients no terminations due to drug related AEs were reported for the remainder of the extended follow-up period after the six terminations due to non-compliance/gastrointestinal (GI) AEs that were observed in the efficacy phase of the trial [5].

## Discussion

Lomitapide, in combination with other lipid-lowering therapies is an effective treatment for the reduction of LDL-C levels in adult patients with HoFH, allowing many of these patients to reach EAS recommended LDL-C target levels for the first time.

Most of the patients who achieved LDL-C targets did so in the first 6 months of treatment. Assessment of continuous maintenance of these LDL-C target levels is difficult in a setting where background lipid-lowering therapies, including apheresis, could be titrated upon achievement of target levels. Similarly, not all patients who achieve target during lomitapide dose titration are likely to continue on the maximum dose reached in the long-term. In our analysis, patients that reached the 100 mg/dL goal at least once, remained at goal for a median of 6 study visits suggesting that many of the patients receiving lomitapide are able to maintain the target long-term. Nevertheless, the data provided here are a reflection of the potential to reach target, rather than an indication of maintenance of LDL-C at target.

The annualized MACE rates in HoFH patients treated with mipomersen, lomitapide and evolocumab were 11.4, 2.0 and 2.1%, respectively. The pre-mipomersen exposure annualized event rate in the mipomersen trial was 26.1%.

We chose the dataset from the publication of Duell, et al. [9] as a comparator as it reports relatively recent outcome data for HoFH patients receiving standard of care therapy before newer agents such as lomitapide, mipomersen and evolocumab became available. We were unable to locate other recent data that would have allowed us to calculate annualized event rates for HoFH patients receiving conventional lipid-lowering therapy.

One of the major limitations of our MACE rate comparison is that the lomitapide studies were open-label and did not include a control group receiving placebo. Similarly, neither the mipomersen nor the evolocumab studies include placebo arms of sufficient duration to assess CV event rates. The background event rate for patients receiving conventional lipid lowering therapy used for this comparison is therefore derived from the pre-treatment mipomersen cohort that differs in important aspects, such as apheresis usage, baseline LDL-C levels, or CVD rates at baseline from the population enrolled in the lomitapide and evolocumab studies. Indeed, the patients in the Duell study had baseline LDL-C values of 455 mg/dL, which is significantly higher than the 336 mg/dL and 324 mg/dL in the lomitapide and evolocumab studies. This may be due to underlying disease severity or due to less intensive background therapy, specifically apheresis (63 and 32% of patients in the lomitapide and evolocumab trials received apheresis at baseline, compared with none in the mipomersen studies). However, baseline LDL-C levels are likely a strong predictor of achievable LDL-C reductions and CV risk and are therefore a significant confounding factor in our analysis. Baseline CVD prevalence is another important potential confounder. In the lomitapide studies baseline CVD prevalence was 93%, it was 51% in the evolocumab study and not reported for mipomersen.

Further limitations of this post-hoc, retrospective analysis include small sample sizes, relatively short treatment durations, and the possible biases inherent in conducting retrospective evaluations of this type. Importantly, the CV event rate reported by Duell et al. [9] could be skewed by subjects experiencing recurrent events (7 patients account for 12 events) or preferential recruitment of subjects with above average cardiovascular risk. Our study also relied on AE reporting for capture of MACE and events were not formally adjudicated. It is therefore possible that we may not have captured all events, or that some events, such as unstable angina, may have been misclassified. Furthermore, not all patients enrolled in the pivotal trial entered the lomitapide extension study, thereby introducing potential bias in the extension trial population towards a healthier ‘survivor’ population. The mipomersen treatment duration was also significantly shorter than the lomitapide exposure, and it is conceivable that longer treatment with mipomersen may have been associated with a progressive lowering of MACE rates. Thus, our findings cannot be regarded as a definitive estimation of MACE reduction with lomitapide, and can also not be used to draw any definitive conclusions about the long-term outcome associated with any one of the novel lipid-lowering therapies. It would have been preferable to combine data from multiple HoFH cohorts to obtain averaged CV event rates in patients receiving conventional lipid-lowering therapy, but we were not able to locate any data that would have allowed us to calculate annualized CV event rates.

Despite major limitations our data, along with the data for mipomersen and evolocumab, suggest that CV risk in HoFH can be reduced by pharmacotherapies that reduce LDL-C. The cohort of patients with the highest LDL-C (pre-mipomersen; LDL-C 455 mg/dL) had the highest event rates. Reducing the LDL-C in this cohort to levels comparable to those found at baseline in the lomitapide and evolocumab studies was associated with an approximate 50% reduction in MACE. The event rate, however, remained higher than that seen with lomitapide and evolocumab, likely because of prior and ongoing increased exposure of the vasculature to LDL. Baseline LDL-C levels were comparable for lomitapide and evolocumab (336 mg/dL and 324 mg/dl) and on-treatment MACE rates were remarkably similar. Although lomitapide lowered LDL-C more than evolocumab, differences in the prevalence of CVD at baseline (93% for lomitapide versus 51% for evolocumab) may have influenced CV event rates. The prognostic importance of LDL-C is reinforced by data from Raal et al., who showed that lowering LDL-C from 615 mg/dl to 452 mg/dL with conventional lipid-lowering therapy was also associated with delayed cardiovascular events and prolonged survival in patients with HoFH [10].

## Conclusions

Lomitapide lowered LDL-C significantly in patients with HoFH allowing many patients to reach EAS recommended targets for the first time. Comparison of MACE event rates among several HoFH cohorts show that cardiovascular event rates correlate with LDL-C. Lomitapide and other novel lipid-lowering therapy may thus improve the prognosis of patients with HoFH.

## Abbreviations

AE: Adverse event
apoB: Apolipoprotein B
CABG: Coronary artery bypass graft
CHMP: Committee for Medicinal Products for Human Use
CV: Cardiovascular
CVD: Cardiovascular disease
EAS: European Atherosclerosis Society
EMA: European Medicines Agency
FDA: Food and Drug Administration
GI: Gastrointestinal
HoFH: Homozygous familial hypercholesterolaemia
LDL-C: Low-density lipoprotein cholesterol
LDLR: LDL receptor
LDLRAP1: LDL-receptor adaptor protein 1
MACE: Major adverse CV events (MACE)
MI: Myocardial infarction
PCSK9: Proprotein convertase subtilisin/kexin type 9
SD: Standard deviation
UA: Unstable angina
